# Drop Jumping on Sand Is Characterized by Lower Power, Higher Rate of Force Development and Larger Knee Joint Range of Motion

**DOI:** 10.3390/jfmk7010017

**Published:** 2022-02-04

**Authors:** George Giatsis, Vassilios Panoutsakopoulos, Iraklis A. Kollias

**Affiliations:** Biomechanics Laboratory, Department of Physical Education and Sport Science at Thessaloniki, Aristotle University of Thessaloniki, 54124 Thessaloniki, Greece; bpanouts@phed.auth.gr (V.P.); hkollias@phed.auth.gr (I.A.K.)

**Keywords:** biomechanical analysis, kinetics, kinematics, stretch shortening cycle, vertical jumping, surface stability, balance, impact

## Abstract

Plyometric training on sand is suggested to result in advanced performance in vertical jumping. However, limited information exists concerning the biomechanics of drop jumps (DJ) on sand. The purpose of the study was to compare the biomechanical parameters of DJs executed on rigid (RIGID) and sand (SAND) surface. Sixteen high level male beach-volleyball players executed DJ from 40 cm on RIGID and SAND. Force- and video-recordings were analyzed to extract the kinetic and kinematic parameters of the DJ. Results of paired-samples *t*-tests revealed that DJ on SAND had significantly (*p* < 0.05) lower jumping height, peak vertical ground reaction force, power, peak leg stiffness and peak ankle flexion angular velocity than RIGID. In addition, DJ on SAND was characterized by significantly (*p* < 0.05) larger rate of force development and knee joint flexion in the downward phase. No differences (*p* > 0.05) were observed for the temporal parameters. The compliance of SAND decreases the efficiency of the mechanisms involved in the optimization of DJ performance. Nevertheless, SAND comprises an exercise surface with less loading during the eccentric phase of the DJ, thus it can be considered as a surface that can offer injury prevention under demands for large energy expenditure.

## 1. Introduction

Sand surfaces (SAND) are a demanding exercise surface. Research evidence suggests that a higher energy cost is required for running [1,2,3,4,5], walking [6,7], sprinting [8,9] and jumping [10,11,12] on SAND compared to rigid (RIGID) surfaces. Despite the higher energy cost, training on SAND causes positive adaptations in key strength and conditioning factors such as aerobic endurance, concentric strength of the leg extensor muscles and agility in a variety of sport disciplines [13].

SAND is recommended for training in volleyball and beach-volleyball (BV) players due to the observed favorable performance adaptations, i.e., the improvement in technique, muscle strength, vertical jump height, agility and endurance [14,15,16,17,18,19]. In particular, BV players improved their jumping ability after the application of training programs on SAND that included jumping exercises utilizing the stretch-shortening cycle (SSC), namely the counter-movement (CMJ) and drop jumps (DJ) [20,21]. However, there is a bias in the literature about the effectiveness of SAND with regard to the facilitation of adaptations in the SSC. The application of training with CMJ and DJ resulted in increased muscle activation of the knee extensor muscles that was interpreted as a positive adaptation in SSC due to SAND [21]. On the opposite, past research findings suggest that, after training on SAND, the improvement in the CMJ jump height was decreased compared to the respective vertical squat jump (SQJ) [10]. In addition, it is suggested that the increased power after the implementation of training on SAND was not a result of an enhanced utilization of SSC [15]. Thus, the effectiveness of SAND for inducing neuromuscular adaptations for the efficient utilization of SSC is questionable [13].

Based on the contemporary knowledge of DJs executed on RIGID, derived from both practice and research, plyometric training enabling the SSC is considered as the best training method to provoke adaptations in key jump performance factors such as force, speed, and power [22]. These adaptations are the reason to include SSC training exercises in volleyball and BV, especially those exercises that require a rapid SSC function [23]. This requirement is fulfilled by executing DJs in a manner that results in an increased power output [24,25,26]. In detail, the increased mechanical power during vertical jumps is related with the regulation of stiffness that occurs during ground contact [27,28]. However, despite the positive adaptations observed in a variety of jump-related biomechanical variables during vertical jump tests, the implementation of plyometric training on SAND in female volleyball players resulted in a non-significant, yet notable, 3.7% decrease in DJ performance [14].

In general, a good vertical jumping performance is of major importance for BV players since vertical jumping is present in the majority of the skills of the sport [29,30,31,32]. However, past research reported differences concerning vertical jumping on SAND compared to RIGID [29,33,34,35,36]. In detail, these studies reported lower jumping heights in vertical jumps executed on SAND. Furthermore, it was reported that elite BV players achieved higher jump heights on RIGID comparing to SAND in SQJ and CMJ by 14% and 15.4%, respectively [35,36]. This is due to the compliance of SAND that increases the demands for energy expenditure in order to execute the vertical jump in an explosive manner [11,12]. In addition, lower force application and power production were reported during the propulsion in SQJ and CMJ [10,29,33,35,36]. A previous kinematical analysis in the above-mentioned vertical jump tests revealed that, in order to overcome the constrains imposed by SAND, the ankle joint is extended faster [35,36]. Thus, the knowledge of the biomechanical differences concerning the key kinetic and kinematic parameters of vertical jumping on SAND and RIGID is of importance for designing efficient training programs aiming the optimization of the SSC for BV players.

Despite the current knowledge of the biomechanics of vertical jumping on SAND, there is a gap in the literature concerning the biomechanics of DJs performed on SAND. The effect of SAND is mainly studied in a pre-post study design that examines vertical jumping on RIGID [14,20]. A number of studies has measured vertical jump performance on SAND using jump and reach tests [19,21,29,31]. Other researchers provided information about jumping on SAND using photocell mats [10], accelerometry [15] and inertial measurement devices [32]. Kinetic and kinematic parameters derived from force-plate data have been reported only for SQJ and CMJ [33,35,36]. To the best of our knowledge, there is limited information regarding DJs on SAND since only the magnitude of the ground reaction forces [34] and ground/flight time [11] have being reported. Thus, further insight is needed for the key kinetic factors (i.e., rate of force development, power, stiffness) that can evaluate the effectiveness of the execution of DJs on SAND.

The purpose of the study was to compare the kinetic and kinematic parameters of DJ executed on RIGID and SAND. It was hypothesized that DJ on SAND will result in lower jumping height, force and power output, as well as larger lower extremity joint range of motion compared to RIGID.

## 2. Materials and Methods

### 2.1. Participants

The minimal number of participants to achieve an effect size of 0.8, power of 0.9 and *a* = 0.05 for the maximum jump height measurement was found to be 15 according to the estimation made using the G*Power v.3.1.9.7 software [37]. Thus, 16 adult professional male BV players (26.2 ± 5.7 y, 1.87 ± 0.05 m, 83.4 ± 5.8 kg) served as participants in the study. Participation was on a voluntary basis and was allowed after obtaining a signed consent. The inclusion criteria were the participation in an international Federation International de Volleyball (FIVB) tournament, to be finalists in BV tournaments included in the national championship calendar, to exhibit records of systematic participation in their training and competition program and to have a competitive experience in BV of at least five years. The exclusion criterion was the incident of an injury or locomotor disability in a period of 6 months prior testing. The measurements took place during the competitive season of the national championship. The study was approved by the Institutional Ethics Committee (approval No.: 87/2021).

### 2.2. Procedure

**Figure 1:**
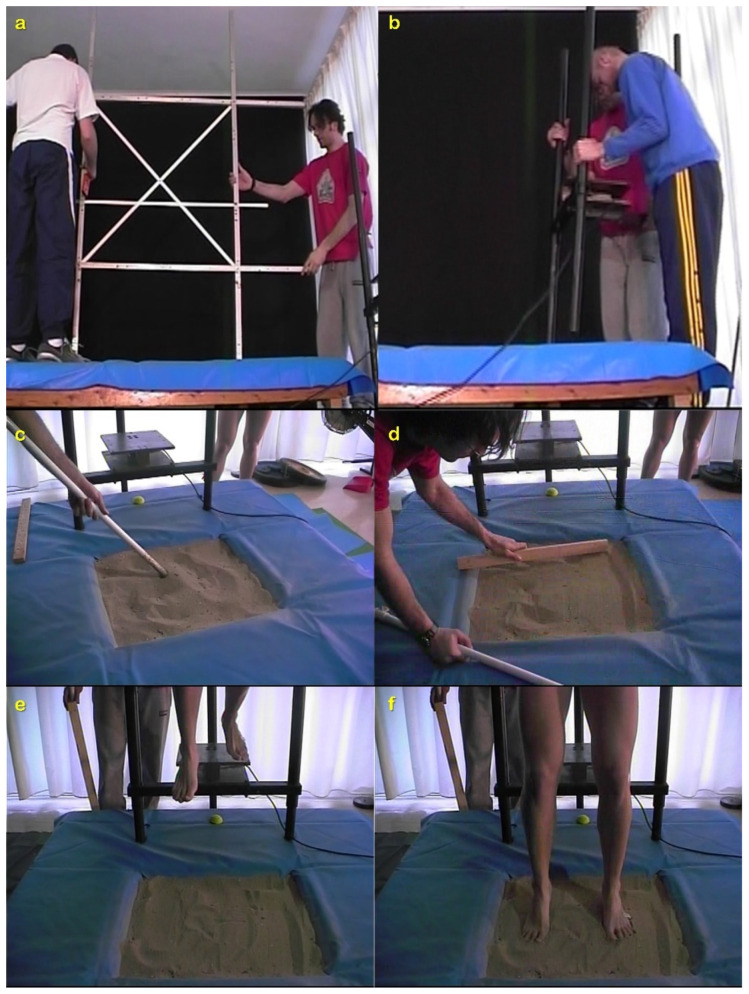
Experimental set-up and procedure for SAND: (**a**) calibration frame placement; (**b**) adjustment of the drop force-plate to the safety platform; (**c**) mixing the sand; (**d**) making the surface even; (**e**) take-off form the drop plate with a roll-off; (**f**) instant of touchdown in the sand pit where the examination for excessive plunging into the SAND was conducted.

SAND was simulated by firmly attaching a wooden sand pit on the force-plate. The wooden pit, with dimensions of the bottom and of the top side equal to 46 × 50 cm and 59 × 63 cm, respectively, while its depth was 31 cm, was constructed to contain the sand particles (Figure 1).

The base dimensions of the sandpit were exactly the same as the embedded to the ground force plate in order to be exactly in contact with each other. Before the actual data acquisition, it was established that the participants’ mass recorded by the ground force plate was exactly the same with and without the sandpit. The edges of the sandpit were covered with soft materials for safety reasons, i.e., if a faulty landing occurred. Additionally, a canvas sheet was placed away from the sandpit and covered the surrounding safety platform that was 116 × 150 × 31 cm (length, width and height, respectively), which was also used to hold inside the sand particles. The total weight of the wooden pit, including the sand, was 120.12 kg. The sand fulfilled the FIVB requirements for the conduction of official BV tournaments. This was established after checking the physical properties and grain size distribution of the sand as determined from a series of laboratory tests according to the American Society for Testing and Materials (ASTM) and that are described in detail elsewhere [36]. Prior each jump, the sand was mixed throughout its volume with a custom tool that was marked at 31 cm to resemble the sandpit height. This tool was used to evenly spread the sand within the wooden sand pit and to avoid compaction of the sand particles.

Warm-up consisted of cycling on an 817E Monark (Exercise AB, Vansbro, Sweden) Cycle-Ergometer, dynamic stretching exercises and sub-maximal vertical jumps. DJs from 40 cm were executed barefooted in a random order on SAND and on RIGID, with the arms kept on the trunk. The dropping height of 40 cm was selected due to past research suggestions [38]. The drop was performed from a custom made one-dimensional force-plate (1-Dynami, ©: Biomechanics Lab AUTh, Thessaloniki, Greece) that was adjusted and fixed within the safety platform (Figure 1). The instructions given to the participants were to “drop with a roll-off” movement [39] and to “jump as high and as fast as possible” [40]. Participants executed three DJs on each surface. During the experimental DJs on SAND, a Redlake Motionscope PCI 1S camera (Redlake Imaging Corporation, Morgan Hill, CA, USA), operating at 250 fps, was used to visually inspect excessive plunging into the sand that resulted in the annulment of the trial. Finally, only the attempt with maximum jump height (h_JUMP_) achieved in each condition was selected for further analysis.

### 2.3. Data Acquisition and Analysis

An AMTI OR6-5-1 force plate (AMTI, Newton, MA, USA) recorded the 3D components of the ground reaction forces (GRF). The sampling frequency for both the force-plates used for data acquisition was set to 500 Hz. The following parameters were calculated [40]:

1. Temporal parameters: total ground contact time (Tc); downward phase duration; time to achieve maximum vertical Ground Reaction Force (tvGRF); time to achieve peak power during the upward phase (tP).
2. Spatial/kinematic parameters: h_JUMP_; body center of mass (BCM) vertical displacement during the downward and upward phases; BCM vertical velocity.
3. Kinetic parameters: GRF vertical, medio-lateral and anterio-posterior component; rate of force development (RFD); work (W); power (P).

The impact velocity of the BCM on the ground was determined using the take-off data from the drop-force plate [41]. Firstly, the initial velocity of the jump (impact velocity after the drop) was calculated from the time-integral of the net force recorded from the drop force-plate. The flying time of the drop was measured from the synchronous data acquisition from both force-plates. Thus, the BCM velocity at the instant of the landing after the drop phase (*U_IMPACT_*) was calculated as shown in Equation (1):

(1) $$U_{IMPACT}=U_{DROP}-g\times t_{FLIGHT}$$

where *U_DROP_* is the BCM velocity at the instant of take-off from the drop force-plate, *g* is the acceleration of gravity and *t_FLIGHT_* is the duration of the drop phase.

Afterwards, h_JUMP_ was calculated using the vertical BCM take-off velocity derived from the integration of the net vGRF. RFD was directly extracted as the first time-derivative of the recorded vGRF. Vertical BCM displacement was extracted through the integration of the vertical BCM velocity. Work was calculated by multiplying vertical BCM displacement with net vGRF and power as the time-derivative of work.

Besides the parameters mentioned above, stiffness parameters were also examined. The vertical stiffness was calculated as the ratio of vertical GRF to vertical BCM displacement and leg stiffness as the ratio of vertical GRF to the change of the leg length [42]. To extract the latter, DJs were also video-recorded at 100 fps with a digital video-camera (JVC GR-DVL 9600 EG, Victor Company of Japan Ltd., Yokohama, Japan). The camera was fixed on a tripod placed 7.6 m from the force plate and at a height of 1.2 m, with the camera axis being perpendicular to the plane of motion. A 2.5 m × 2.5 m calibration frame was also recorded to conduct a 2D-DLT analysis for the extraction of the 2D coordinates and the angular kinematics of the lower limb joints [35]. The examined angular kinematic parameters were the ankle, knee and hip range of motion (ROM) and the respective peak angular velocity (ω) of the lower limb joints during the downward and upward phases.

### 2.4. Statistical Analyses

The Kolmogorov–Smirnov (*p* > 0.05) and the Levene’s test (*p* < 0.05) were used to establish the existence of normal distribution and equality of variance of the data, respectively. The results of these tests validated the use of Paired-Samples *t*-test to check possible significant differences between RIGID and SAND. Effect sizes were estimated after calculating Cohen’s *d* (≤0.49 = small, 0.50–0.79 = medium, ≥0.80 = large) [43]. All statistical tests were conducted using the IBM SPSS Statistics v.27.0.1.0 software (International Business Machines Corp., Armonk, NY, USA), with the level of significance set at *a* = 0.05 for all statistical analyses.

## 3. Results

The results for the spatiotemporal parameters are presented in Table 1. No significant differences (*p* > 0.05) were observed in the examined parameters except for h_JUMP_, which was lower in SAND (medium effect size).

The vertical displacement of the BCM was almost identical on either surface for both the downward and upward phase. Significant (*p* < 0.05) differences were observed for the majority of the examined kinetic parameters in the upward but not in the braking phase (Table 2). In detail, larger peak vertical GRF was recorded in RIGID compared to SAND (small effect size). No differences (*p* > 0.05) were observed for the other two components of GRF. RFD was significantly (*p* < 0.05) larger in the downward phase of SAND than in RIGID (large effect size). Power at the downward phase was almost equal between surfaces, but a significantly (*p* < 0.05) larger power output was observed in RIGID compared to SAND in the upward phase (medium effect size). Significantly (*p* < 0.05) lower work was observed both in the downward and upward phase of SAND compared to RIGID (large and medium effect size, respectively). Regarding stiffness, no significant difference (*p* > 0.05) was found for vertical stiffness due to the surface. On the opposite, peak leg stiffness was significantly (*p* < 0.05) lower in SAND than RIGID (medium effect size).

**Figure 2:**
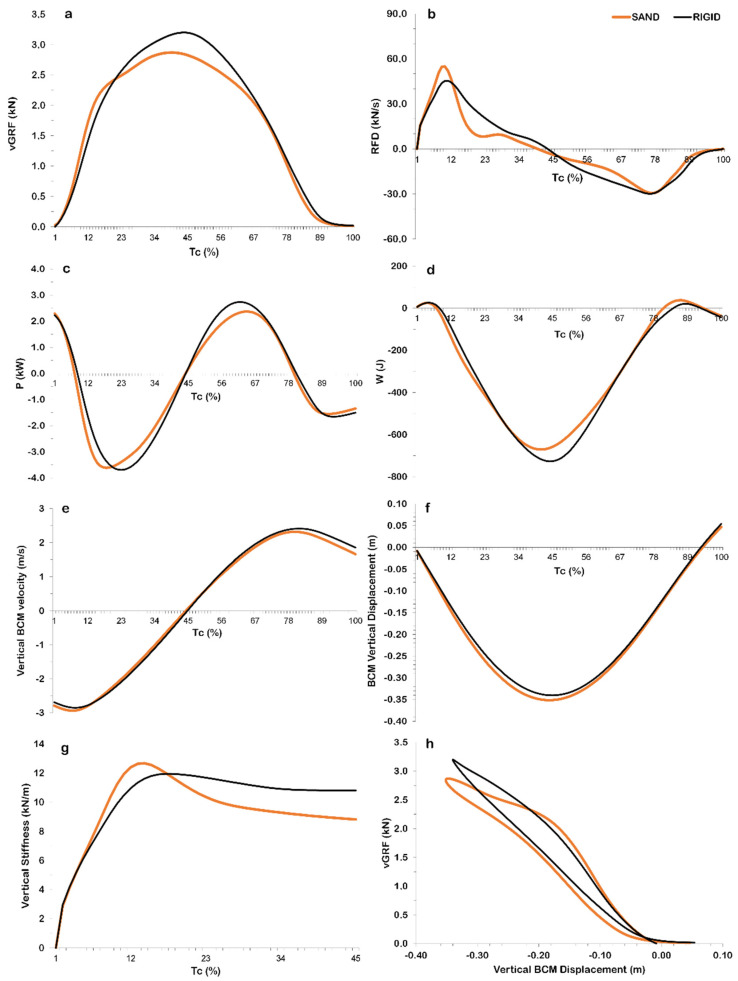
Mean ensemble (*n* = 16) time−history curves for the examined drop jump kinetic parameters on rigid (RIGID) and sand (SAND) surface: (**a**) vertical ground reaction force; (**b**) rate of force development; (**c**) power; (**d**) work; (**e**) body center of mass vertical velocity; (**f**) body center of mass vertical displacement (0 = body center of mass height at the instant of touchdown); (**g**) vertical stiffness; (**h**) vertical stiffness depicted by plotting the vertical body center of mass displacement vs. the vertical ground reaction force. Abbreviations: vGRF: vertical Ground Reaction Force; RFD: Rate of Force Development; P: power; W: work; BCM: body center of mass; Tc: contact time. NOTE: all curves are normalized with respect to Tc; the curves in Figure 2h are depicted for the time period from touchdown to the lowest height of the BCM during the contact with the surface.

The qualitative examination of the time–history curves of the kinetic parameters revealed almost identical patterns between surfaces (Figure 2). Minor alterations were noted for RFD and vertical stiffness. For the former, a steeper peak was revealed during the initial stage of the downward phase in SAND compared to RIGID (Figure 2b). Vertical stiffness in RIGID exhibited a plateau after reaching its peak value, as for SAND the peak value was of a larger magnitude with respect to the following plateau (Figure 2g).

No significant (*p* > 0.05) differences were observed in the majority of the examined lower limb joint angular kinematical parameters during the downward phase (Table 3). However, a significant difference (*p* < 0.05) was observed for the knee joint range of motion that was larger in SAND compared to RIGID (large effect size). The peak angular velocity of the ankle joint was also significantly different (*p* < 0.05), since it was smaller in SAND than in RIGID (medium effect size). No significant (*p* > 0.05) differences were found in the upward phase.

**Figure 3:**
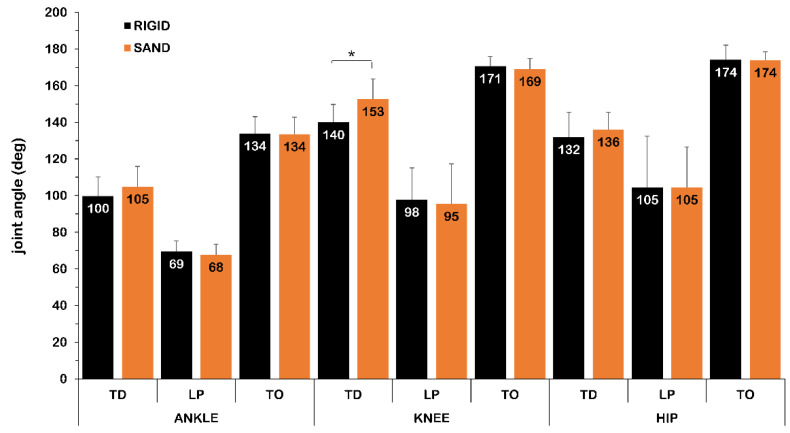
Joint angles at the instants of touchdown (TD), lowest vertical position of the body center of mass (LP) and take-off (TO) of the drop jumps on rigid (RIGID) and sand (SAND) surface (*n* = 16; *: *p* < 0.05).

The examination of the joint angles at specific instances of the DJ, namely the touchdown, the lowest vertical position of the BCM and the take-off revealed a significant (*p* < 0.05) difference for the knee joint angle at touchdown (Figure 3). In specific, the knee joint was about 13 degrees more extended at touchdown in SAND than in RIGID (*t*_1,15_ = −4.202, *p* = 0.001, *d* = 1.23; large effect size).

No significant difference (*p* > 0.05) was revealed for the knee joint at the selected instances of the DJ. In addition, no significant differences (*p* > 0.05) were evident for the ankle and hip joint angles.

**Figure 4:**
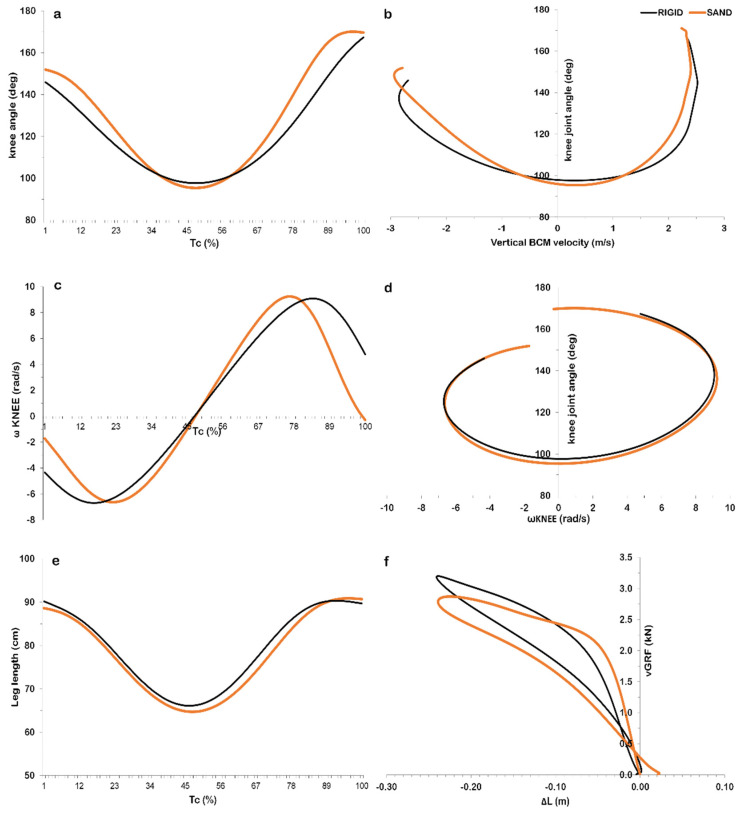
Mean ensemble (*n* = 16) time–history curves for the examined drop jump kinetic parameters on rigid (RIGID) and sand (SAND) surface: (**a**) knee joint angle; (**b**) knee joint angle with respect to the body center of mass vertical velocity; (**c**) angular velocity of the knee joint; (**d**) knee joint angle with respect to the angular velocity of the knee joint; (**e**) leg length; (**f**) change of leg length (0 = leg length at the instant of touchdown from the drop phase) with respect to the vertical Ground Reaction Force. Abbreviations: Tc: contact time; BCM: body center of mass; ωKNEE: angular velocity of the knee joint; ΔL: change of leg length; vGRF: vertical Ground Reaction Force. NOTE: curves depicted in Figure 4a,c,e are normalized with respect to Tc.

The qualitative examination of the time–history curves of the examined angular kinematic parameters also revealed almost identical patterns between surfaces (Figure 4). Minor alterations were noted for the knee joint angle and the angular velocity approximately at the first 30% and at the last 30% of the ground contact phase of the DJ (Figure 4a,c). The deceleration of the body was accompanied with a more rapid knee joint flexion at the downward phase in SAND (Figure 4b). In the upward phase, the knee joint extended to its take-off angular position earlier in SAND than in RIGID (Figure 4d). Regarding leg stiffness (Figure 4f), a similar plateau, in terms of the respective plateau observed for vertical stiffness (Figure 2h), was revealed in the downward phase for SAND.

## 4. Discussion

The aim of the study was to compare the biomechanical parameters of DJ executed on SAND and RIGID. Results revealed that DJ on SAND had lower jumping height (−19.8%), force and power output, as well as larger RFD, work, knee ROM and peak ankle angular velocity at the downward phase than RIGID. Thus, the hypothesis of the study was confirmed.

DJ jump height was lower on SAND than RIGID, being in line with past research results concerning the comparison of SQJ and CMJ on different surfaces [11,29,33,34,35,36,45,46]. The ground contact time was in considerable agreement with past findings [47,48]. It is commonly agreed among researchers that the lower jumping heights observed in the vertical SQJ and CMJ tests are caused by the lower force and power outputs observed for SAND compared to RIGID [33,35,36]. In the present study, power was significantly lower in the upward phase. Thus, the lower jump height for SAND can be explained by the lower power output, since power is suggested to be a determinant factor for the optimization of DJ performance [40,48,49,50,51,52,53]. A possible reason for not achieving larger power in SAND can be attributed to the fact that SAND is an unstable surface and inhibits the fast application of force during jumping [29,35,54]. In addition, as depicted in Figure 4, a more rapid knee joint flexion was at the downward phase in SAND. This finding, in combination with the lower angular velocity of the ankle flexion, reveals a different pattern to negotiate the deceleration of the body due to the different stiffness of the surface to execute the jump.

Unlike previous observations in SQJ [35] and CMJ [36], the time to achieve maximum vertical GRF on SAND was not different compared to RIGID. This finding, combined with the larger RFD during the downward phase in SAND indicates that ground contact with the sand was highly unstable. This led the participants to make a strenuous effort to overcome these constraints that were imposed for the execution of the jumping task. However, the medio-lateral and anterio-posterior components of GRF were not different between surfaces. Thus, there is an indirect indication that the balance requirements at the initial phase of the DJ did not differ between surfaces. Nevertheless, it is suggested that the deformation of SAND increases the requirements for dynamic stability [6,12,17,55]. Additionally, SAND comprises a demanding surface to execute explosive movements since its surface is characterized by larger friction compared to other sport surfaces [33]. The interaction with SAND during exercise utilizing the SSC is suggested to absorb large amounts of energy [3,10,11,29,33,34]. In addition, jumping on SAND utilizing the SSC is proposed to lead to lower re-use of the stored elastic energy [10]. These factors eventually result in increased work expenditure. Furthermore, recent research evidence suggests the existence of an additional protective neuromuscular mechanism when “dealing” with landings on harder, less “safe” surfaces, guaranteed even by visual input alone [56].

In the present study, less negative work was done in the downward phase in SAND compared to RIGID. This could be an indirect indicator of a lower rate of energy absorption during the downward phase in SAND [38]. Nevertheless, the lesser negative work could be associated with smaller amounts of elastic energy stored in the series elastic elements and eventually with the lower jumping height in SAND [10,38]. Thus, due to the observed bias, this point has to be further investigated in future studies examining DJ on SAND.

The importance of the knee joint biomechanics as a regulator of DJ performance has been highlighted in past research [57]. resent data revealed that the knee joint extended to its take-off angular position earlier in SAND than RIGID. Thus, less power was generated about the knee joint that could be a cause for the lower DJ performance found in SAND. Power, besides suggested to be a determining factor for DJ performance, is related with changes in vertical stiffness after plyometric training [58]. Furthermore, the mechanical power produced during the upward phase of a DJ is maximized when an optimal leg stiffness occurs in the downward phase [25,27]. In the present study, leg stiffness was lower in SAND. This is not in agreement with past research reporting that equal leg stiffness can be exhibited when performing a DJ on surfaces with different compliance [59]. Leg stiffness is affected by the knee and ankle stiffness [60]. In the present study, both joints showed significant differences concerning their angular kinematics. This finding could be related with the significantly larger peak leg stiffness observed in RIGID. Nevertheless, a notable maintenance of relatively constant values regarding leg stiffness was observed in both RIGID and SAND, confirming past findings [44,59]. In general, lower stiffness during landing is proposed to be related with mechanisms of long-term adaptations caused by eccentric exercise aiming to prevent injuries [61,62]. In conclusion, larger stiffness is related with the inability to resist large eccentric loadings [63], which seems to be the case for RIGID.

Compared to RIGID, DJ on SAND was executed with a more extended knee joint at the instant of impact. In addition, during the downward phase, the knee joint range of motion was larger, while the ankle joint flexed with a slower angular velocity. This combination was reported in the past for DJs executed from higher compared to lower dropping heights [38] and could be considered as a protective mechanism to avoid excessive loading. In addition, in the present study, no differences were observed in the upward phase between the examined surfaces for the lower limb joint angular kinematics. This is not in alignment with past research concerning running or sprinting on SAND, where a backward movement of the feet due to the deformation of the sand at the end of the push off is common [1,8,10]. This might be the result of the relatively long duration of the ground contact time, during which SAND could have been compressed at the downward phase. This could eventually lead the sand surface to dissipate some of its absorptive qualities and thus resemble a more rigid surface [36]. The present finding also cannot support research evidence which suggested that performing SSC on SAND is related to the muscle action during the propulsive phase that resulted by the compensation made in the braking phase in terms of the degradation of elastic energy [10,13]. However, previous studies for the CMJ on SAND reported a significant effect of surface for the knee [45] and ankle joint kinematics [36]. The connection between the two aforementioned joints is the biarticular gastrocnemius muscle, which can affect the ankle range of motion and can cause differences in key biomechanical factors of DJ, such as RFD and knee angular kinematics [64]. It has been proposed that the common adaptation of the neuromuscular system to deal with the differences caused by the instable SAND surface is to exhibit a higher co-contraction of the lower limb muscles, which eventually results in a less optimum flow of energy [1]. However, it was found that muscle activity and muscle–tendon unit mechanical properties of the gastrocnemius muscle increase when jumping from a deformable surface. This led to the conclusion that “internal regulatory mechanisms exist to compensate for differences in surface properties” [65]. Both of the above factors should be further investigated in the future.

Training on SAND was found to be effective for improving sprinting, jumping and balance ability of team sport players [14,20,66,67,68], and that there is a significant association between specific agility and vertical jump tests on SAND [46]. Additionally, the adaptations of jumping, sprinting and agility were found to be transferred on RIGID [13,15,16]. One reason is the increased motor unit recruitment after the implementation of plyometric training on SAND [21]. In general, performing exercises on SAND is suggested to reduce the musculoskeletal loading in training and rehabilitation programs [69,70]. SAND training is proposed for preseason training due to the decreased muscle soreness, faster recovery and the lower probability of overuse injuries [1,55,71]. Larger training adaptations are expected since heavier training loads can be implemented on SAND [10,56]. However, due to the lower stiffness of SAND, there are limited neuromuscular adaptations since the mechanical stimuli on the musculoskeletal system are reduced. Thus, SAND is less effective for the improvement of explosive movements [10,13,72]. The results of the present biomechanical analysis of DJ on SAND seem to confirm the above notion.

The findings of the present study should be considered given its limitations. The recording of muscle activation patterns during DJ could provide further insight concerning the examination of the regulation of stiffness. Another possible limitation is the fact that only one drop height was selected for analysis; thus, the present results cannot be generalized to interpret DJ on SAND and should be read with caution. Nevertheless, the present study revealed an insight regarding the biomechanics of DJ on SAND that provides information for a widely used jumping modality in training practice and testing environments. It is of importance that the participants in the study were top-level BV players which had extensive training experience in DJ on both SAND and RIGID. Thus, the comparison of DJ biomechanical parameters between the examined surfaces can be considered to be reliable. The present findings are of interest to coaches and researchers, particularly under the perspective of the kinetic and kinematic differences of executing the drop jump on a rigid and on a sand surface.

## 5. Conclusions

The compliance of the SAND seems to decrease the efficiency of the mechanisms involved in optimizing the DJ performance compared to RIGID. Nevertheless, SAND comprises an exercise surface that imposes a lesser load during the eccentric phase of the DJ compared to RIGID. Therefore, SAND can be used in jumping programs aimed at injury prevention or for rehabilitation programs after an injury in lower extremities. In addition, due to the highly unstable surface of SAND, participants were found to increase knee joint range of motion during the downward phase to fulfill the locomotor requirements to execute the jumping task and to acquire the necessary stability to do so. Finally, due to the higher energy expenditure required on SAND, DJs can be used in the pre- or off- training season not only in beach volleyball, but also in other team and individual sports that include jumping activities in their technique.

## Figures and Tables

**Table 1:** Means ± standard deviations of the comparison for the spatiotemporal parameters of the drop jumps on RIGID and SAND surface (*n* = 16).

Parameter	RIGID	SAND	*MD*	*SE*	*t*	*p*	*d*
Center of Mass displacement (cm)							
Jump height (h_JUMP_)	27.9 ± 4.2	24.4 ± 4.8	3.5	0.9	3.933	0.001 *	0.78
Downward phase	−33.8 ± 12.2	−33.9 ± 8.8	0.1	2.0	0.031	0.976	0.01
Upward phase	39.3 ± 12.6	38.6 ± 12.6	0.6	0.2	0.319	0.754	0.06
Temporal (ms)							
Contact time	408.4 ± 135.5	430.4 ± 121.3	22.0	15.8	1.396	0.183	0.17
Downward time	186.1 ± 72.8	192.0 ± 60.1	5.9	8.7	0.673	0.511	0.09
tvGRF	175.3 ± 82.4	155.3 ± 51.9	19.9	22.1	0.901	0.382	0.29
tP	280.9 ± 126.2	294.8 ± 110.5	13.9	14.2	0.977	0.344	0.12

*: *p* < 0.05; *MD*: mean difference; *SE*: standard error of the mean; h_JUMP_: jump height; tvGRF_:_ time to achieve maximum vertical Ground Reaction Force; tP: time to achieve maximum power during the upward phase.

**Table 2:** Means ± standard deviations of the comparison for the kinetic parameters of the drop jumps on RIGID and SAND surface (*n* = 16).

Parameter	RIGID	SAND	*MD*	*SE*	*t*	*p*	*d*
Peak Ground Reaction Force (kN)							
Vertical (vGRF; net force)	2.48 ± 0.84	2.14 ± 0.56	0.43	0.15	2.359	0.032 *	0.48
Anterior–Posterior (xGRF)	0.36 ± 0.05	0.37 ± 0.08	0.01	0.03	0.205	0.841	0.15
Mediolateral (yGRF)	0.11 ± 0.04	0.11 ± 0.04	0.01	0.01	0.498	0.627	0.14
Peak Rate of Force Development (kN/s)							
Downward phase	−53.3 ± 14.0	−71.6 ± 25.1	18.3	5.6	3.248	0.005 *	0.90
Upward phase	44.0 ± 11.6	40.1 ± 6.7	3.8	2.6	1.471	0.161	0.41
Peak Power (kW)							
Downward phase	−4.2 ± 1.2	−4.3 ± 1.2	0.1	0.2	0.776	0.289	0.08
Upward phase	3.1 ± 1.0	2.6 ± 0.6	0.5	0.2	2.245	0.040 *	0.61
Peak Work (J)							
Downward phase	−738.4 ± 110.7	−662.6 ±89.2	75.8	21.5	3.518	0.003 *	1.36
Upward phase	778.1 ± 98.6	713.3 ± 86.3	64.8	26.6	2.535	0.023 *	0.70
Stiffness (kN/m)							
Peak Vertical stiffness	11.6 ± 4.0	12.6 ± 3.9	0.8	0.8	1.061	0.305	0.25
Peak Leg stiffness	8.6 ± 4.9	5.1 ± 3.8	3.5	1.5	2.367	0.032 *	0.79
Average Leg stiffness	3.8 ± 2.9	4.0 ± 3.9	0.2	0.9	0.198	0.846	0.06

*: *p* < 0.05; *MD*: mean difference; *SE*: standard error of the mean. Leg stiffness parameters are according to Struzik and Zawadzki [44].

**Table 3:** Means ± standard deviations of the comparison for the joint kinematic parameters of the drop jumps on RIGID and SAND surface (*n* = 16).

Parameter	RIGID	SAND	*MD*	*SE*	*t*	*p*	*d*
*Downward phase*							
ROM_ANKLE_	30.30 ± 10.69	37.16 ± 12.51	6.86	3.94	1.742	0.102	0.59
ROM_KNEE_	42.20 ± 16.84	57.21 ± 14.85	15.01	3.79	3.965	0.001 *	0.95
ROM_HIP_	23.93 ± 26.37	31.52 ± 18.78	7.59	5.86	1.294	0.215	0.33
ω_ANKLE_	−6.12 ± 1.74	−5.03 ± 1.76	1.09	0.50	2.168	0.047 *	0.62
ω_KNEE_	−7.38 ± 1.45	−7.15 ± 0.88	0.23	0.39	0.582	0.569	0.19
ω_HIP_	−4.05 ± 1.92	−4.23 ± 1.51	0.19	0.37	0.499	0.625	0.10
*Upward phase*							
ROM_ANKLE_	64.40 ± 11.66	65.84 ± 11.27	1.44	2.02	0.713	0.487	0.13
ROM_KNEE_	72.93 ± 17.14	73.65 ± 14.96	0.72	2.90	0.248	0.807	0.05
ROM_HIP_	66.13 ± 25.18	69.33 ± 18.98	3.20	5.04	0.634	0.536	0.14
ω_ANKLE_	10.24 ± 1.82	9.62 ± 1.21	0.63	0.36	1.735	0.103	0.40
ω_KNEE_	10.16 ± 1.29	10.27 ± 0.72	0.11	0.26	0.410	0.687	0.11
ω_HIP_	8.41 ± 1.13	8.14 ± 0.98	0.27	0.23	1.164	0.263	0.26

*: *p* < 0.05; *MD*: mean difference; *SE*: standard error of the mean; ROM: joint range of motion (in degrees); ω: angular velocity (in rad/s).

## Data Availability

The data that were acquired and analyzed in the present study are available from the corresponding author upon reasonable request.
